# Evaluation the elemental, micromorphological and microhardness changes in dentin after removal of caries with sodium hypochlorite-based or enzyme-based chemomechanical caries removal agents: an in vitro study

**DOI:** 10.1186/s12903-025-07479-w

**Published:** 2025-12-29

**Authors:** Mahmoud S. Ahmed, Maha M. Ebaya, Hamdy H. Hamama

**Affiliations:** Conservative Dentistry Department, Faculty of Dentistry, Mansoura University, Mansoura, Egypt

**Keywords:** Chemomechanical caries removal, Papain, SEM, Micromorphological analysis

## Abstract

**Background:**

This study was conducted to evaluate the elemental, micromorphological and microhardness changes in dentin after the removal of caries with sodium hypochlorite-based or enzyme-based chemomechanical caries removal (CMCR) agents.

**Methods:**

Twenty-one cavitated carious molars and 7 noncarious permanent molars were used. The samples were split into four groups: group (1): traditional rotary technique; group (2): enzyme-based CMCR agent; group (3): NaOCl-based CMCR agent; and group (4): control (sound). After caries excavation, all the samples were subjected to elemental analysis via energy dispersive X-ray (EDX) spectroscopy, micromorphological analysis by scanning electron microscopy and, finally, Vickers microhardness testing. Elemental analysis results were evaluated via one-way ANOVA followed by Tukey’s HSD and Bonferroni post hoc comparisons. Vickers hardness values were analyzed via two-way ANOVA followed by Tukey’s HSD post hoc multiple comparison evaluations.

**Results:**

Elemental analysis revealed no variation in Ca levels (*p* > 0.05) among the four groups. The results revealed a variation (*p* < 0.05) in P levels between the conventional rotary and control sound groups and the enzyme-based and NaOCl-based groups. The SEM results revealed occluded and partially occluded dentinal tubules and the presence of a smear layer following rotary caries removal and revealed patent dentinal tubules with minimal or absent smear layers following enzyme-based caries removal. Finally, there were patent dentinal tubules and a small smear layer following NaOCl-based caries removal. The Vickers hardness of sound dentin and remaining dentin after rotary caries removal was greater (*p* < 0.05) than the Vickers hardness of the remaining dentin after CMCR by enzyme-based or NaOCl-based agents, and no variation (*p* > 0.05) in the Vickers hardness of the remaining dentin was observed between the enzyme-based and NaOCl-based groups.

**Conclusions:**

CMCR methods are conservative substitutes for rotary excavation and have translational potential for maintaining the repair capability of the dentin-pulp complex following clinical caries excavation. Compared with the NaOCl-based caries excavation technique, the Papain-based enzymatic CMCR agent appears to be a conservative substitute for traditional rotary excavation of carious tissue, and this enzymatic technique improved the morphological characteristics of the remaining dentin for further bonding. Unlike the conventional method and sound dentin, the mineral composition of dentin was affected by the two CMCR agents.

**Supplementary Information:**

The online version contains supplementary material available at 10.1186/s12903-025-07479-w.

## Introduction

Dental caries is the most prevalent non-communicable disease globally; the World Health Organization (WHO) estimates that it affects approximately 2.5 billion people [1]. Cavitation occurs after several months or years of this process; the final stage of the disease process is known as dental caries [2]. Dental caries, a highly predominant disease, is regarded as a serious oral wellness issue. Caries detection, prevention, and treatment approaches have evolved over time because of the reversible, dynamic nature of the disease [3]. Deep carious lesions of the dentin are managed more conservatively and biologically [4], since traditional rotary excavation is associated with the vibratory motion, high temperatures, and removal of both healthy and diseased tissue nonselectively [5, 6]. 

Dental researchers are looking for suitable minimally invasive (MI) procedures to alleviate the limitations of bur excavation. To remove the outermost, bacterially infected, and denatured dentin while conserving the partly demineralized tissue that may be restored with appropriate rehabilitative restoration [7]. This maintains the quality of the sound and mineralizable dental structure while increasing the healing capability of the dentin_pulp complex [8]. These techniques involve laser ablation, air abrasion, sonoabrasion, and chemomechanical caries removal agents (CMCRs).

CMCR is one of the most protective methods for caries excavation, employing minimally invasive caries removal procedures [9]. In 1976, Goldman introduced the adoption of a solution of chemical substances to eliminate dental caries. These procedures rely primarily on the selective removal of caries-unrepairable dentin, hence keeping caries-affected tissue untouched. The fundamental concept of the CMCR method revolves around changing the chemical structure of the carious tooth, which softens it and then removes it manually with a hand tool [10, 11]. 

These strategies are necessary to offer additional infection control measures since they do not generate unwanted aerosols or particles that are trapped within the air. This often leads to various transmissible viruses, notably our newest epidemic coronavirus (COVID-19), which predominantly propagate throughout the respiratory system via droplets, secretions from the airway, and contact with the skin [12]. CMCR agents are classified into a pair of groups: sodium hypochlorite-based agents (NaOCl) and enzymatic-based agents (papain-enzymatic-based CMCR agents) [13]. 

The papain-based enzymatic CMCR agent employed in this study is a national production agent that is patentable, authorized, and recognized in Brazil. Its main ingredients are papain, chloramine and toluidine blue [14]. It has several benefits, including simple application, a waiting period of 30–60 s after the application of no demand for specific tools to administer it, and no requirement for local anesthetic equipment, with the incorporation of drilling [15, 16]. Systematic reviews indicate that papain-based CMCR agents offer significant advantages in patient comfort and reduced need for anesthesia compared to rotary methods [17, 18]. Furthermore, a recent systematic review of in vitro studies confirms that chemomechanical agents can offer a less invasive alternative, often resulting in dentin surfaces with more open tubules and less smear layer [19].

The sodium hypochlorite-based CMCR agent (Cariemove, DM Trust Company, Egypt) used in this study represents a newer formulation. Unlike traditional sodium hypochlorite solutions, its composition includes amino acids and carboxyglycine in addition to sodium hypochlorite and excipients. This combination is designed to create a buffered, potentially less aggressive oxidative environment. The proposed mechanism involves the oxidative breakdown of denatured collagen by hypochlorite, while the added organic compounds may help to moderate the reaction and improve the gel’s chemomechanical debriding efficacy. However, as a relatively new commercial product, independent data on its effects on the dentin substrate remain limited.

The evaluation of micromorphological and microhardness changes in dentin following CMCR is critical for predicting the clinical performance of subsequent adhesive restorations. Previous in vitro studies have provided foundational insights: SEM analyses consistently reveal that papain-based agents tend to produce dentin surfaces with patent tubules and minimal smear layer [20, 21], whereas NaOCl-based agents may leave a partially occluded tubule structure with a variable smear layer presence [22, 23]. Concurrently, microhardness testing often indicates that dentin remaining after CMCR is softer than that after rotary excavation, reflecting the preservation of the deeper, caries-affected dentin layer [20, 24]. These analyses are indispensable; SEM elucidates the surface topography critical for resin hybridization and micromechanical retention, while surface microhardness (SMH) provides an indirect measure of the dentin’s mechanical properties and mineral density, which are key indicators of its health and ability to support a restoration [25].

The rationale for comparing these two types of CMCR agents lies in their fundamentally different mechanisms of action. Papain-based agents function through proteolytic digestion of denatured collagen, while NaOCl-based agents act via oxidative dissolution. This difference may lead to distinct alterations in the dentin substrate, potentially influencing its bonding characteristics and the long-term prognosis of restorations. A direct comparative evaluation of their effects on elemental composition, micromorphology, and microhardness is therefore clinically relevant to inform material selection. Furthermore, despite the documented patient benefits of CMCR techniques, a comprehensive in vitro comparison between this specific newer NaOCl-based gel and an established papain-based gel is lacking.

Therefore, this study was conducted to evaluate and compare the elemental, micromorphological and microhardness changes in the dentin of healthy teeth (control group) and after the removal of caries with NaOCl-based or enzyme-based CMCR agents and the conventional rotary method. The subsequent null hypotheses were that the three caries excavation strategies (conventional rotary method, enzyme-based CMCR and NaOCl-based CMCR) and the control treatment did not significantly affect the Ca or phosphorus levels in the dentin. There was no substantial change in dentin morphology or microhardness among the three excavation techniques and the control group.

## Materials and methods

### Materials

The current investigation used two commercially accessible chemomechanical caries removal products: an enzyme-based chemomechanical caries removal agent (Papacarie, Formula & Acao, Brazil) and a sodium hypochlorite-based chemomechanical caries removal agent (Cariemove, DM Trust Company, Egypt). Each material was employed following the manufacturer’s specifications. The standard rotary procedure was carried out via a low-speed handpiece (NSK, Tochigi, Japan) at 35,000 rpm utilizing a round steel bur (#31, MANI, Japan) after adding a caries detector dye. (Cario finder, TOOTHMATE, Egypt) as described in Table 1.

**Table 1:** Materials used in the study

Material	Type	Manufacturer	Composition	LOT	Application method
Papacarie	Enzyme-based CMCR agent	Formula&Acao, Brazil	Papain enzyme, chloramine, toluidine blue, salts, preservatives, a thickener, stabilizers, deionized water	9637	(1) Apply gel to the carious lesion. (2) Allow it to sit for 30–60 s until bubbling is observed. (3) Gently remove the softened carious dentin with a spoon excavator without pressure. (4) Reapply fresh gel and repeat the process until the gel remains clear and the dentin feels hard to probing. (5) Rinse the cavity with water and wipe with a moist cotton pellet.
Cariemove	NaOCl-based CMCR agent	DM Trust Company, Egypt	Amino acids, sodium hypochlorite carboxyglycine and excipients	DMH1316	(1) Apply the purple gel to the carious lesion. (2) Allow it to sit for 30 s to initiate the chemical action. (3) Remove the softened caries with a sharp spoon excavator. (4) Thoroughly irrigate the cavity with air and water spray.5. Repeat the procedure for shorter durations (15 s) until all non-repairable carious dentin is removed
Cariofinder	Caries detector dye	TOOTHMATE, Egypt	Polyethylene glycol, methylene blue dye and base material	03A2023	(1) After initial excavation, rinse and dry the cavity. (2) Apply the dye with a microbrush, leave for 10 s. (3) Rinse thoroughly with water. (4) Remove any stained (non-repairable) dentin with a bur.5. Repeat until no further staining occurs.
Low-speed handpiece	**- -**	NSK, Tochigi, Japan		**-**	
Round steel bur	**- -**	(BR-31) - MANI, Japan			

### Sample size calculation

The sample size was determined on the basis of the microhardness variations in dentin upon caries excavation via sodium hypochlorite-based or enzyme-based chemomechanical caries removal agents retrieved from previous research (Hamama et al., 2013). The total estimated sample size will be at least 7 in each group utilizing the G power program version 3.1.9.7 to analyze the sample size according to an effect size of 2.17,2-tailed test, α error = 0.05, and power = 95.0%.

### Tooth selection

Twenty-one cavitated carious molars and 7 recently extracted, non-carious permanent human molars were collected from healthy people. Periodontal disease was the primary cause of the extraction. The collection and use of human teeth for this in vitro study were approved by the Ethical Review Committee of Faculty of Dentistry, Mansoura University under approval number A14061222. All procedures were performed in accordance with the relevant guidelines and regulations, including the Helsinki Declaration. Teeth were collected with prior informed consent from patients who were seeking dental care at the Oral and Maxillofacial Surgery Department Clinic, Faculty of Dentistry, Mansoura University. Teeth were collected following the infection control guidelinesTeeth were collected following the infection control guidelines. After any remaining soft tissue from the teeth was removed via a hand scaler (Zeffiro, Lascod, Florence, Italy), the teeth were preserved for 72 h in a disinfectant solution (0.5% chloramine-T) [19]. The selected teeth were polished with a rubber cup and pumice water slurry. The teeth were preserved in distilled water (including a 0.1% thymol solution), which was changed every 5 days throughout the study and removed only during the test to avoid dehydration [20]. 

The selection of carious teeth was made after periapical X-rays on the teeth were taken to detect the degree of caries; these teeth were inspected radiographically upon mounting on an acrylic jaw model. A radiograph was taken via cone beam digital radiography (paralleling technique); the film encompassed the coronal, middle, and apical thirds of the teeth. The International Caries Classification and Management System (ICCMS) states that teeth in stage 5 that were clinically cavitated and had radiolucency that extended to the innermost third of the dentin were selected.

### Study design and grouping system

Four groups of seven teeth each were randomly selected from the sample (*n* = 7):

Group (1): Conventional caries excavation method and caries detector dye; group (2): Enzyme-based CMCR agent; group (3): NaOCl-based CMCR agent; and group (4): Control (sound). For the first 3 groups, widening of the enamel was performed before excavation of the caries.

For Group 1, the conventional rotary method, caries excavation was accomplished with a low-speed handpiece (NSK, Tochigi, Japan) at a speed of 35,000 rpm with water cooling, using a round steel bur (BR-31, ISO 001/018, MANI, Japan). This speed represents a standard low speed recommended for selective caries removal to minimize pulpal irritation and provide better tactile control, consistent with established protocols [22]. A caries detector dye (Cario finder, TOOTHMATE, Egypt) was applied to characterize the carious lesion and ensure the elimination of caries-unrepairable dentin, leaving behind caries-repairable dentin. The carious dentin was subsequently washed and dried, the caries detector dye was dispensed into a deep dish (1_2 drops), and a small sponge or a mini-brush was used for the application. After the carious dentin was completely covered and rubbed gently, the cavity was finally rinsed with water following a 10-second pause. Unrepairable carious dentin clearly exhibited deep blue staining. A sharp steel bur was used for caries unrepairable dentin removal. After the cavity was rinsed with water, a strong air blast was used to dry it for further inspection of caries unrepairable dentin, and the procedures were repeated until caries unrepairable dentin removal was complete.

For group 2, an enzyme-based chemomechanical caries removal agent, papacarie gel, was used to treat the carious cavity (Formula & Acao, Brazil). The gel was applied to the carious lesion which was allowed to sit for 30 s. Oxygen was released following the breakdown of collagen, causing bubbles to form on the surface, which means that the removal process started. Next, the carious lesion was carefully scratched away with a spoon excavator devoid of pressure. At the excavation site, more fresh gel was placed until the gel became clear. After the gel was removed, the cavity was cleared and wiped with a wet cotton pellet.

**Fig. 1:**
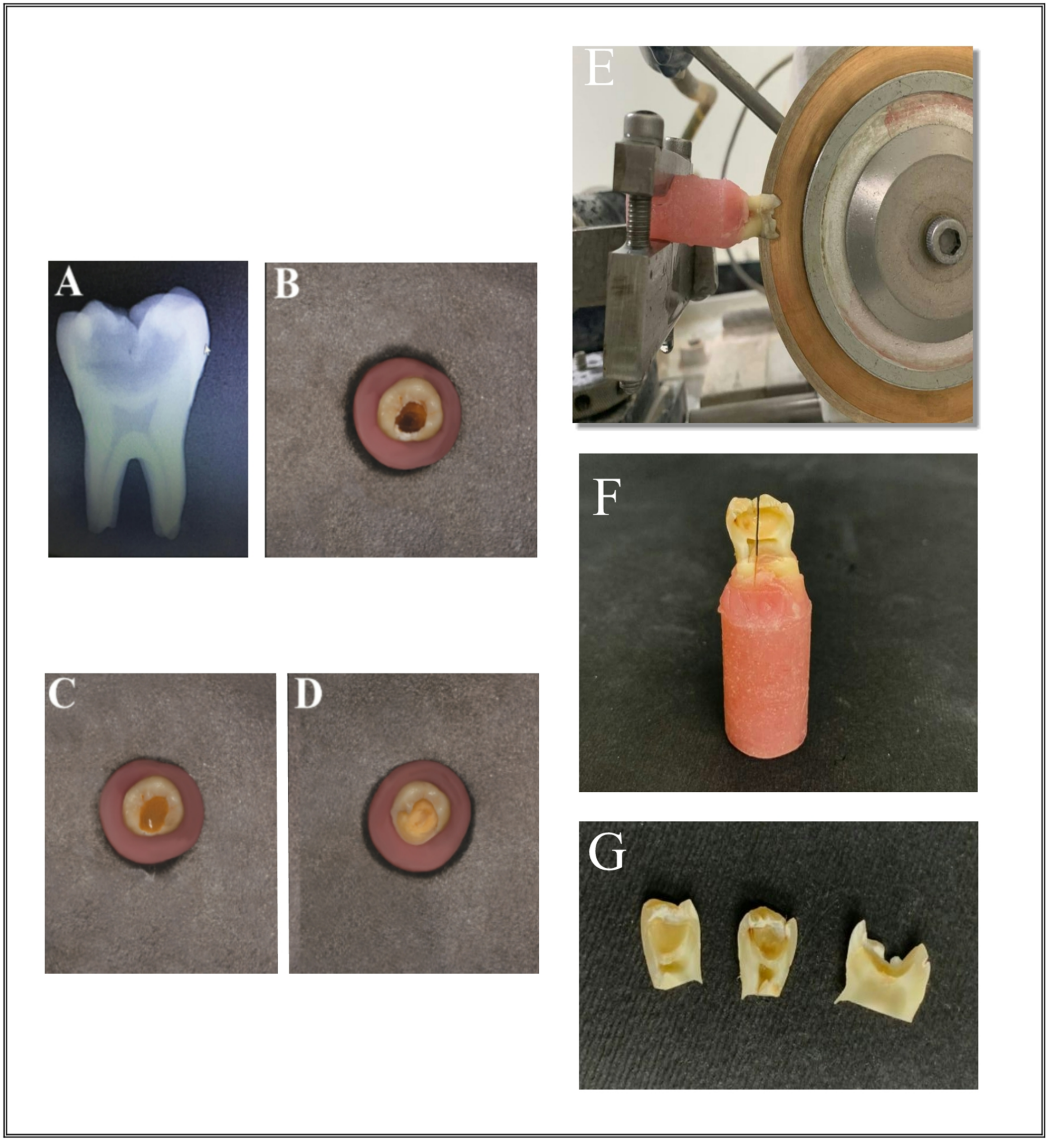
Caries excavation: **A** Cone beam digital radiography was used to take a radiograph (paralleling technique) and the radiolucency extending to the innermost 1/3 of dentin, **B** Occlusally cavitated carious molar, **C** Carious lesion covered with NaOCl-based CMCR agent (cariemove), **D** Post-excavation view of the lesion site. A specimen preparation: **E** Mesiodistal cutting for a specimen across the carious lesion with a low-speed water-cooled diamond saw (Isomet, Buehler Ltd, Lake Bluff, IL, USA), **F** A buccolingual direction cutting via the middle of the excavated caries lesion into 2 sections, one for EDX and the other for micromorphological scanning with an electron microscope, **G** A separated molar before doing the tests

Group 3 involved the use of a chemomechanical caries removal agent based on NaOCl; cariemove gel (DM Trust Company, Egypt) was used to treat the carious lesions. After the gel was applied to the carious lesion, it was allowed to sit for 30 s. A sharp spoon excavator was subsequently utilized to eliminate the carious lesion. The cavity was then irrigated thoroughly with air and water. All procedures were repeated for shorter durations (15 s) until all carious unrepairable dentin was removed, as shown in Fig. 1.

Finally, Group 4 was the control group, as it was composed of 7 caries-free teeth and was stored in distilled water, which was changed every 5 days until use.

### Specimen preparation

**Fig. 2:**
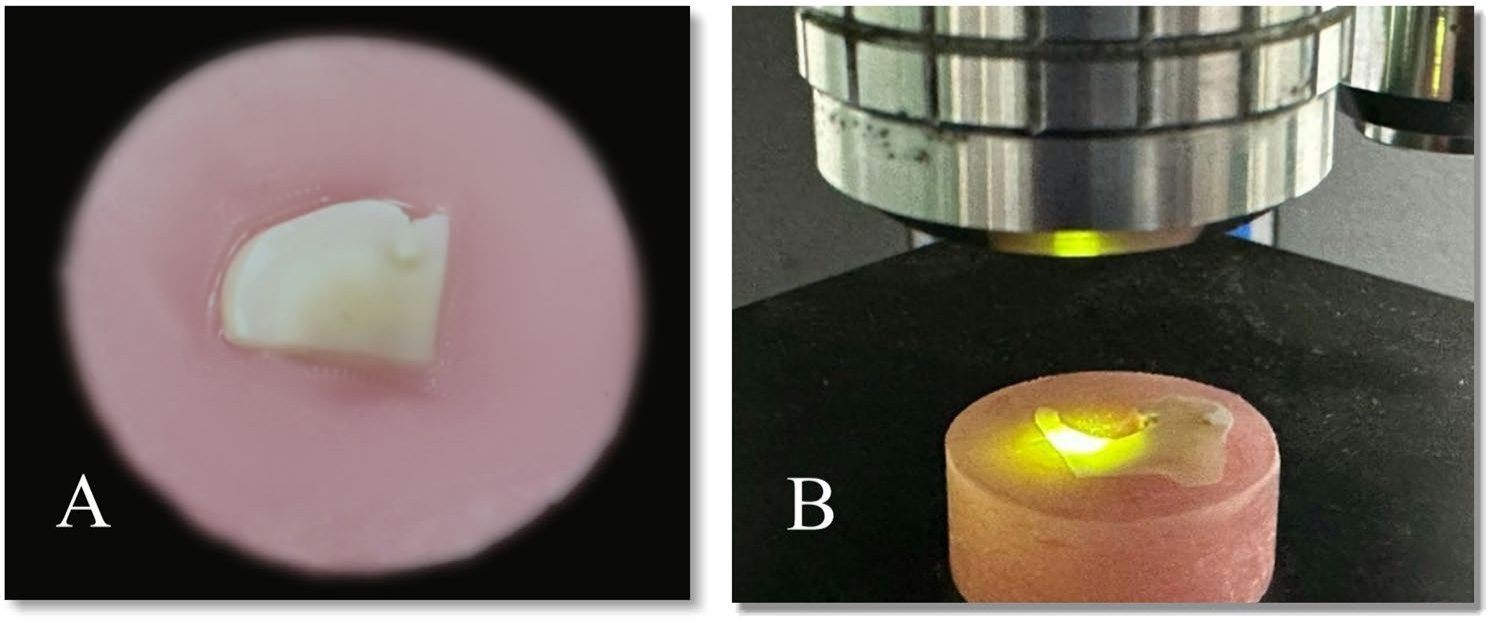
**A** A specimen embedded horizontally in acrylic resin blocks that the buccal and lingual surfaces are flat because the indentation of the microhardness tester requires a flat surface, **B** An indentation by the diamond micro indenter

For the first 3 groups, after caries removal and examination with a dental loup with a magnification of 3.5X to ensure that there was no exposure site, the 28 molars were carefully sliced at the cementoenamel interface utilizing a low-speed diamond saw (Isomet, Buehler Ltd., Lake Bluff, IL, USA), as shown in Fig. 1. The crowns were set in acrylic blocks, and every single tooth was cut into two equal parts longitudinally in the mesiodistal plane across the carious lesion with a low-speed water-cooled diamond saw. Individual samples were submerged in self-curing acrylic resin blocks (Duralay; Reliance Dental C., Worth, IL, USA) in specifically produced plastic molds with an inner diameter of 10 mm and a height of 20 mm, with the dentin surface facing upward, as shown in Fig. 2.

All the samples were exposed to ultrasonic vibration in a digital ultrasonic bath (Guilin, Woodpecker, Guangxi, China) with deionized water for five minutes to ensure that all the debris had been removed from the tooth surface. The first half was divided into two halves by cutting through the middle of the excavated caries lesion in the buccolingual direction, as shown in Fig. 1. One portion was then utilized for elemental analysis via energy dispersive X-ray spectroscopy (EDX), whereas the other portion was examined for micromorphology by scanning electron microscopy. The second half was evaluated for microhardness.

For the control group, as before after cleaning and polishing, the root of each molar was sectioned and discarded, while the crown was sectioned mesiodistally into two halves. Then, the first half was cut in the buccolingual direction into two sections; one section was used for elemental analysis with EDX, and the other section was evaluated for micromorphological analysis by scanning electron microscopy. The second half was evaluated via a microhardness test.

The samples were tested for surface mineral content, surface micromorphology and surface microhardness at the flattened dentin surface.

### Elemental analysis of dentin via an energy dispersive x-ray system (EDX)

The acrylic blocks of the samples were removed after the application of separating medium. The samples were left in a dry atmosphere and then mounted on aluminum studs [21]. The first section’s longitudinal flat cut surface was coated with a gold sputter and then subjected to elemental analysis via an energy dispersive X-ray system (EDX) under high vacuum at 1500X magnification (Microanalysis suites, INCA V4.11, Oxford Instrument Analytical Ltd, UK), which was connected to a scanning electron microscope (Hitachi S-3400 N, Hitachi High Technologies, America, Inc. Schaumburg, IL, USA). The X-ray beam was focused on the intertubular dentin at three separate locations, approximately 50 μm from the edge of the excavation site’s deepest part, to perform the chemical analysis. The average recorded mineral values are presented, with Ca and P being the most important minerals at these three points [22]. 

### Micromorphological analysis of dentin

The excavated surface of the residual part was chosen for micromorphological investigation via field emission scanning electron microscopy (FESEM) (JEOL, Tokyo, Japan). Prior to the treatment, the samples were placed in a hot air oven for 15 min to ensure total dehydration and then mounted on aluminum studs [23]. The samples were sputtered with gold via a coater technique. They were then evaluated via an electronic probe microanalyzer (JOEL-EX-840)- with a 10 mm working distance and a 30 kV accelerating voltage [24]. The study area was scanned at 1000x magnification for each sample, and the most instructive images were saved for SEM analysis.

### Microhardness test

The surface microhardness (SMH) of the samples was evaluated via a digital microhardness tester, the Tukon 1102 Wilson hardness tester (BUEHLER Germany), with a Vickers elongated diamond pyramid indenter under a weight of 100 g for 10 s. The samples were implanted in self-cured acrylic resin (ProBase ColdTM; Ivoclar Vivadent AG, Liechtenstein). To prevent dentin dehydration during the implantation procedure during setting, the sample surfaces were allowed to remain revealed and concealed with a moist fiberless laboratory napkin (KimwipesTM Ex-L, Kimberly_Clark Professional, USA). Following the setting, the surfaces of every portion were wet ground with silicon carbide papers (800, 1200, 2400, and 4000 grit) (MicrocutTM, Buehler, USA) to thoroughly expose and flatten the dentin surface [22]. 

The indentations were measured with precision microscopes. The Vickers hardness number (VHN) was measured by producing three indentations in various parts of each sample and calculating their average to reflect the hardness value of the sample [25]. The indentations were performed one mm apart from one another to minimize the residual stress. Surface microhardness testing was performed on all the samples at 50 μm, 100 μm, and 150 μm distances from the floor of the excavated lesion. This process produced an indentation that was clearly defined as shown in Fig. 2, as the main requirements for verifying that an outline was clearly indented and the absence of defects in the area to be measured in the tooth [26]. All the readings were carried out by the same examiner using the same calibrated machine. The following formula was used to determine the SMH values: HV equals 1.854 P/d2. where P is the load in kgf, d is the diagonal length in millimeters, and HV is the Vickers hardness in kgf/mm2.

### Statistical analysis

The extracted data were input into a Microsoft Excel sheet and analyzed via the Statistical Package of Social Science (SPSS) software (V.26, IBM, NY, USA).

## Results

### Elemental analysis outcome

Table 2 displays the EDX elemental analysis of Groups 1 through 4. One-way ANOVA revealed that the Ca levels of the four groups did not vary greatly (*p* > 0.05). There was also a significant difference (*p* < 0.05) in P levels between the “Papain-based, NaOCl-based” and “Sound, Rotary” groups. Tukey’s test revealed these differences, indicating that there was no substantial difference (*p* > 0.05) between the sound and rotary groups and that there was no substantial difference (*p* > 0.05) between the papain-based and NaOCl-based groups.

**Table 2:** Results of elemental content of sound and remaining dentin following caries removal

Group	*N*	Ca (mean ± SD)	*P* (mean ± SD)
Sound	7	0.29 ± 0.01^A^	0.15 ± 0.01^a^
Rotary	7	0.29 ± 0.02^A^	0.15 ± 0.01^a^
Papain-based	7	0.28 ± 0.02^A^	0.14 ± 0.01^b^
NaOCl-based	7	0.27 ± 0.01^A^	0.14 ± 0.01^b^

^A^ Groups with similar superscripted upper-case letters within the same column show no significant differences

^a, b^ Groups with similar superscripted lower-case letters within the same row show no significant difference

### Micromorphological analysis (SEM)

**Fig. 3:**
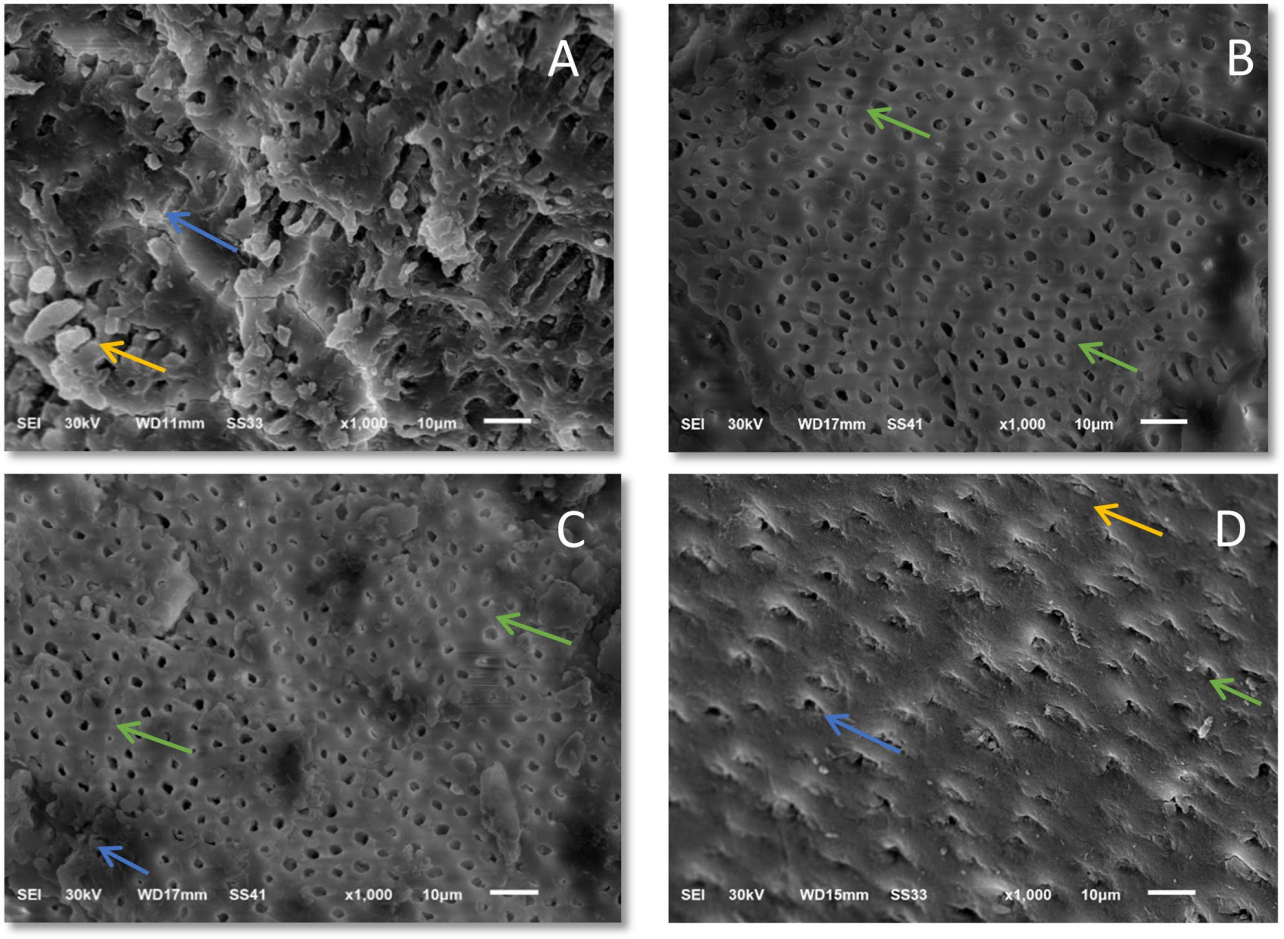
**A** SEM micrograph in low (X 1000) Magnification of remaining dentin following rotary caries treatment shows blocked and partially occluded dentinal tubules and the presence of a smear layer, **B** SEM micrograph in low (X 1000) Magnification of remaining dentin following papain-based caries treatment reveals patent dentinal tubules with little or no smear layer developed, **C** SEM micrograph in low (X 1000) Magnification of remaining dentin after NaOCl-based caries treatment reveals many and patent dentinal tubules and sparse smear layer, **D** SEM micrograph in low (X 1000) Magnification of remaining dentin for control( sound dentin) shows open and occluded dentinal tubules and the presence of a smear layer. Green arrow: shows patent/opened dentinal tubules, Orange arrow shows occluded dentinal tubules, blue arrow shows smear layer

SEM observations were performed at high magnification (×1000); this magnification was selected based on a pilot study that tested different magnifications. Representative SEM micrographs showing the morphological properties of the dentin surface at different intervals and in each treatment group are shown in Fig. 3.

Following rotary caries excavation, the SEM image of the dentin surface consistently revealed the appearance of a homogeneous smear layer created with the round bur, in which cutting anomalies and smear debris covered the tissue with smear plugs. In addition, blockage and no opening of the majority of the dentinal tubules and a smooth, flat dentin surface with a few microirregularities were observed (Fig. 3).

The most notable observation was made in the papain-based group, where the majority of the dentinal tubules were patent, and the smear layer was almost completely absent from every sample and had a rough dentin surface and many microirregularities (Fig. 3).

The remaining dentin in the NaOCl-based group had rough surfaces with increased microirregularities, was patent, had many partially occluded dentinal tubules, and had one or two tubule openings and a partially absent packed smear layer (Fig. 3). In contrast to the rotary method, the remaining dentin surfaces after chemomechanical caries removal via either the NaOCl-based or Papain-based technique appeared more irregular.

### Surface microhardness results

After caries is removed using rotary, papain-based, and naocl-based techniques, the mean Vickers hardness values of healthy dentin (negative control group) and remaining dentin are shown in Table 3. Two-way ANOVA revealed an important impact of the caries excavation method (*p* < 0.05) and distance from the excavated lesion floor (*p* < 0.05) on the dentin Vickers hardness. Furthermore, the interaction of the two variables had a substantial effect on the Vickers microhardness (*p* < 0.05). Tukey’s post hoc test revealed that rotary caries removal resulted in considerably greater Vickers hardness values for sound and remaining dentin (*p* < 0.05) than CMCR by papain-based or NaOCl-based methods. The study revealed that sound dentin had considerably greater Vickers hardness than residual dentin after rotary caries removal (*p* < 0.05) at the 50 μm and 100 μm indentation levels, but no noteworthy distinction was identified at the 150 μm indentation (*p* >0.05). However, there was no substantial difference (*p* >0.05) in the Vickers hardness of residual dentin between the Papain-based and NaOCl-based groups at 50 μm, 100 μm, and 150 μm.

**Table 3:** Results of microhardness of sound and remaining dentin following caries removal

Method	Dentin Vickers Hardness (Mean ± SD)		
	50 μm	100 μm	150 μm
Sound	32.90 ± 0.58^a^	32.53 ± 0.51^a^	32.47 ± 0.38^a^
Rotary	25.44 ± 0.92^c^	27.76 ± 0.58^b^	33.30 ± 1.29^a^
Papain-based	17.14 ± 0.54^f^	20.43 ± 0.93^e^	22.66 ± 0.44^d^
NaOCl-based	16.33 ± 0.84^f^	19.73 ± 0.36^e^	22.44 ± 0.78^d^

^a, b,c, d,e, f^ Groups with similar superscripted lower-case letters within the same row have no significant differences

An additional Tukey’s post hoc test was carried out to examine the influence of the distance from the floor of the excavated lesion on the Vickers hardness of the dentin. Tukey’s post hoc test revealed that the Vickers hardness of the healthy dentin group was not considerably different (*p* > 0.05) across all the indentation locations. In the rotary group, the Vickers hardness of dentin at the 50 μm indentation was considerably lower than that at the remainder of the indentation sites (*p* < 0.05), whereas the Vickers hardness of dentin at the 150 μm indentation was considerably greater than that at the sustaining indentation sites (*p* < 0.05). Compared with those in the 100 μm and 150 μm indentation groups, the Vickers hardness of the dentin in the two papain-based and NaOCl-based groups was substantially lower (*p* < 0.05). The dentin hardness was substantially greater at the 150 μm indentation location than at the other sites (*p* < 0.05).

## Discussion

The present in vitro study evaluated the elemental, micromorphological and microhardness alterations in dentin after caries removal with enzyme-based or NaOCl-based CMCR agents. Although in vivo studies are considered the gold standard method for evaluating the clinical performance of CMCR agents, performing in vitro studies is a significant method for testing new CMCR agents prior to clinical application [27]. Moreover, this study provides the basis for recommendations on how clinicians should use CMCR agents during their daily work [22]. 

Rotary cutting methods can cause problems such as pressure, heat generation, and vibration from the bur’s rotation, adverse biological reactions in the pulp and the nonselective removal of both unrepairable and sound dental tissues, which are particularly unpleasant for patients [28]. This study focused on elevating new caries removal strategies, introduced for dental treatment that aim to overcome these problems. These new methods provide a basis for MICRT development [29]. 

Papain-based CMCR was used in this study, as the efficient removal of infected tissue by the papain enzyme can be attributed to the absence of a plasmatic antiprotase known as antitrypsin. The mechanism of action of papain is believed to involve the degradation of partially degraded collagen molecules, facilitating the disintegration and elimination of the fibrin mantle produced during the carious process while preserving the integrity of intact collagen fibrils. Consequently, the unrepairable dentin undergoes a process of softening, enabling its removal without anesthesia and the use of noncutting instruments [30, 31]. Consequently, the unrepairable dentin undergoes a process of softening, enabling its removal without anesthesia and the use of noncutting instruments. The resulting smear-layer-free surface with patent tubules, as observed in our study, has been shown to enhance the bonding performance of self-etch adhesives to dentin substrates [4, 14].

A CMCR agent based on NaOCl was employed in the present study and contains amino acids, the sodium hypochlorite carboxyglycine and excipients. It is a new experimental agent that needs more studies, and the available data regarding this subject matter are restricted and rely primarily on the instructions provided by the manufacturer. Unlike conventional sodium hypochlorite solutions, this new formulation (Cariemove) includes amino acids and carboxyglycine, which are proposed to buffer its oxidative action and potentially improve its chemomechanical debriding efficacy. The specific role of these additives in moderating the aggressiveness of NaOCl on the dentin substrate warrants further investigation.

In the present study, extracted human teeth were recommended because they mimic oral conditions [32], which was in accordance with previous studies [33, 34]. Teeth were kept in a 0.1% thymol solution prior to the study to avoid any fungal or bacterial growth [35]. The samples were embedded horizontally in acrylic resin blocks such that the buccal and lingual surfaces were flat because the indentation of the microhardness tester required a flat surface [36]. 

The EDX software ‘area selection’ tool was used in this study because it is widely regarded as a reliable approach for identifying subtle fluctuations in mineral composition among closely adjacent zones, such as the superficial and subsurface dentin or hybrid layers [22]. The mineral composition within limited tissue areas can be effectively studied via elemental analysis, a theoretical approach grounded in mathematical methodology. This method is characterized by its conservative nature, nondestructiveness, and simplicity. To determine the elemental composition of the material interface, EDX mapping was employed [37]. 

SEM analysis is also considered the most efficient method for the determination of surface changes in dentin and the presence of a smear layer [22]. Moreover, this analysis has proven to be an adequate, reliable, nondestructive and highly discriminative assessment method for the evaluation of these surface changes [38]. SEM analysis requires coating the samples with metals such as gold, as in the current study, to enhance the image quality [39]. 

The microhardness test was chosen for this study because it is widely used, comparatively easy to use, and consistently yields satisfactory microhardness results [40]. One of the most popular tests for determining the mechanical characteristics and structural integrity of substrates or materials is the Vickers microhardness test. This simple and nondestructive method requires only a small portion of the sample to be examined. A pyramidal-diamond indenter is used to imprint the sample surface for a predetermined amount of time at a specific force [41]. Following the disposal of the load, the size of the impression is assessed under an optical microscope by measuring the size of the diagonal imprint [42]. For the hardness indentation in this study, a weight of 100 g was utilized since it resulted in longer Vickers diagonals, which are recommended to prevent optical measurement errors [26]. 

The mineral content of normal dentine in this study was determined via EDX in earlier studies [43, 44]. The observed levels of phosphorus and calcium were 13 and 27.1, respectively [45]. The EDX findings of the control healthy dentin group this investigation fell within the previously published range [45, 46]. However, in another investigation, EDX revealed a greater Ca/P ratio than did normal dentin because it was a remineralized tissue [47]. In addition, the mineral composition of dentin was affected by the two CMCR agents, unlike the conventional rotary method and the sound dentin, as there was notable variation (*p* < 0.05) in P levels between the “Sound, Rotary” and “Enzyme-based, NaOCl-based” groups. In contrast, prior research has shown that neither of the CMCR compounds had any effect on the mineral composition of dentin [48, 49]. 

This investigation revealed no notable variation (*p* >0.05) in Ca levels among the four groups. This agreed with Hamama HH et al. [22] Chemical changes in carious dentin following the use of CMCR agents based on papain and NaOCl in combination with the traditional rotary method have been reported. Hence, the null hypothesis based on this was acknowledged here, as there was no notable variation in the Ca level of dentin following the three techniques of caries excavation. In another study, Ibrahim LA et al. [50] compared the efficiency of Dual Rinse with NaOCl to that of NaOCl combined with a finishing rinse with 17% EDTA solution. The inorganic chemical structure of the treated dentin in both of the test groups was examined via EDX analysis. Fewer calcium peaks were observed in the dual rinse sample than in the other samples according to EDX analysis.

In addition, this investigation revealed notable variation (*p* < 0.05) in P levels among the four groups, which was not consistent with the findings of Hamama HH et al. [22] When the traditional rotary approach was contrasted with chemical alterations in carious dentin after the use of CMCR agents based on papain and NaOCl, EDX analysis demonstrated no notable variation in P levels among these methods and sound teeth, possibly because of the use of new experimental NaOCl-based agents in our investigation. This substantial variation in the P level did not agree with the finding of Katirci G et al. [51] Additionally, when the chemical composition of the remaining dentin remaining at the cavity bottom following the removal of carious dentin via the NaOCl-based CMCR and ER: YAG laser caries excavation methods was compared with that of the conventional tungsten-carbide bur excavation methods, no notable variation was discovered between the quantities of P content of the cavities treated by the three techniques, possibly because various procedures and materials were utilized between the two studies. Therefore, the null hypothesis was rejected here because of the substantial variation in the P level of dentin among the four groups.

The SEM observations in this study correlated with those of prior studies indicating that the dentin post rotary caries removal was smooth, flat, and coated with a smear layer that occluded the dentinal tubules [44, 52]. When no preconditioning of the substrate was used, the rotary excavation method caused visible smearing, which may disrupt the adherence, wetting, and penetration of adhesive-based restorations [53]. However, the existence of these microirregularities may increase the surface area for micromechanical retention. This finding fits with Hamama et al. [22] Prabhakar et al. noted the existence of a properly formed smear layer with occluded dentinal tubules.

SEM revealed that applying a papain-based CMCR agent caused a dentin surface featuring the absence of a smear layer, leaving patent dentinal tubules. The lack of a smear layer can be explained by the proteolytic nature of the papain gel, as it eliminates the surface caries-infected tissue [54, 55]. This finding is supported by recent studies on deproteinization, which confirm that papain enzyme effectively removes the smear layer and improves the bonding performance of self-etch adhesives to dentin [4, 16]. This dentin surface, which is free of the smear layer, could improve bonding through assisting the infiltration of adhesive resin to reach the intertubular and patent dentinal tubules. Furthermore, the roughened dentin surface related to CMCR techniques may increase the adhesion of the restorative materials owing to the existence of microirregularities, which expand the surface area for bonding [45]. 

This agreed with Hamama HH et al. [22] The morphological differences in carious dentin after the application of a papain-based CMCR agent and the conventional rotary method were compared. Morphological analysis via SEM releaved that the use of a papain-based CMCR caused the dentin surface to be devoid of a smear layer and patent tubules. This agreed with the findings of Arora R et al. [56] The efficiencies of three caries removal techniques, namely, Papain-based, calcium hydroxide and rotary instrumentation, were compared via SEM analyses of the micromorphology of the remaining dentin and resin tags at the resin_dentin interface. The results revealed that Papain-based methods produced few smear layers and open dentinal tubules.

Although the SEM analysis of the NaOCl-based group in this study agreed with the majority of the results of other studies [31, 44, 45, 52], it did not coincide with that of Hossain et al., [45] who stated that NaOCl-based treatment would completely eliminate the smear layer, revealing patent dentinal tubules. They reported that the amorphous layer that obliterated the dentinal tubules was due to the crushing and burnishing of excavated tissue produced through the applicator tip over the surface of the dentin. Thus, the null hypothesis was disregarded here since there was a substantial change in the morphology of dentin after the three excavation approaches.

The observed micromorphological differences between the two CMCR agents have significant implications for their subsequent interaction with adhesive materials. The papain-based agent produced a surface devoid of a smear layer with patent tubules, as confirmed in previous studies [4, 16, 54, 55]. This deproteinized surface is ideal for micromechanical interlocking and resin tag formation, potentially promoting superior bond strength of self-etch adhesives [4, 14]. Conversely, the NaOCl-based agent left a partially occluded tubule structure with a residual smear layer. The oxidative nature of NaOCl is a well-documented inhibitor of free-radical polymerization in resin-based materials [2]. Therefore, despite the potential for micromechanical retention, the residual oxidants from this agent could potentially compromise the bonding efficiency of adhesive restorations by interfering with the polymerization of resin monomers. Further studies directly measuring the bond strength of adhesives to dentin treated with this new NaOCl-based agent are crucial to confirm this effect.

The microhardness test in this study revealed that the remaining dentin after CMCR removal was softer, leading to enormous variation in hardness as opposed to residual dentin after rotary caries removal. Consequently, the null hypothesis was rejected. The caries-repairable dentin, which has lower hardness values, is left behind after the caries-unrepairable dentin is selectively removed, which could account for the reduction in hardness observed in the papain- and NaOCl-based groups [57, 58]. The present study confirmed the findings of Magalhaes et al. [59] who reported that, after conventional caries treatment, the remaining dentin had a higher microhardness than residual dentin with CMCR.

Furthermore, the results of this study agreed with those of Hamama HH et al. [22] The microhardness differences in carious dentin after the use of NaOCl-based or papain-based CMCR agents and the conventional rotary technique were compared. The Vickers hardness of dentin from the groups with caries excavated via the NaOCl-based and papain-based methods was significantly lower than that of the groups with caries excavated via the rotary method and healthy teeth. Conversely, data suggest that the chemomechanical approach yields dentin with hardness values greater than those obtained via the conventional mechanical technique and comparable to those of sound dentin [60]. Additionally, Katirci G et al. [51] compared the microindentation hardness of the dentin that remained at the bottom of the cavity after carious dentin was removed via the Er: YAG laser caries excavation method and the NaOCl-based CMCR agent with that of the traditional tungsten-carbide bur excavation technique. They reported that, compared with samples obtained from conventional and laser excavations, a greater percentage of samples with residual carious dentin were found following NaOCl-based CMCR excavation. The reason here was the use of different techniques, such as Er: YAG lasers and different materials, and Knoop hardness, whereas in our study, Vickers hardness was applied. In another study, Lima Santos et al. [61] did not distinguish the microhardness between rotary excavation and CMCR agents because they employed a different papain-based agent in their investigation.

The microhardness test findings demonstrated that there was no variation in sound dentin hardness throughout the various indentation spots of the cut dentin surface. In a comparable manner, the hardness of the remaining dentin after rotary caries removal showed small differences among the several indentation spots from the floor of the excavated surface. Consequently, as there was no obvious distinction between these sites, the null hypothesis was adopted in this instance. This tends to suggest that excavation via the traditional rotary method eliminates dentin that is both caries-repairable and unrepairable, leaving sound dentin behind. This conclusion was substantiated by the chromatic scale result, which demonstrated that the dentin surface tinted pale pink upon the use of caries detector dye after rotary caries removal. This finding was also verified in a prior study, which revealed that the application of a caries detector dye typically contributes to overpreparation of cavities owing to a lack of specificity of the dye to damaged collagen fibers of the unrepairable dentin, leading to staining of demineralized caries-repairable dentin [43, 49, 62]

The results of the present study are restricted by the evaluation of CMCR agents under laboratory conditions (in vitro studies). The dynamic nature of the variables observed in the oral cavity, including salivary flow characteristics, the existence of plaque, oral hygiene, and the patient’s dietary habits, which might contribute to outcomes that may vary compared with those established in the present study. In addition, a study was carried out to examine the impact of CMCR agents after short periods. Finally, based on the present findings, future studies are recommended to investigate the long-term bond strength and microtensile bond failure modes of adhesive restorations to dentin treated with these CMCR agents. Furthermore, exploring the efficacy of antioxidant agents (e.g., sulfinate salts) in reversing the potential inhibitory effect of NaOCl-based agents on resin polymerization would be of significant clinical value.

## Conclusions

CMCR methods are conservative substitutes for rotary excavation and have translational potential for maintaining the repair capability of the dentin-pulp complex following clinical caries excavation. Compared with the NaOCl- based technique, the Papain-based enzymatic CMCR agent appear to be a conservative substitute for traditional rotary excavation of carious tissue, and this enzymatic technique improved the morphological characteristics of the remaining dentin for further bonding. Unlike the conventional method and sound dentin, the mineral composition of dentin was affected by the two CMCR agents.

## Supplementary Information

Supplementary Material 1.

## Data Availability

The datasets used and/or analyzed during the current study are available from the corresponding author upon reasonable request.
