# Supplementary figures and images for: Evaluation the elemental, micromorphological and microhardness changes in dentin after removal of caries with sodium hypochlorite-based or enzyme-based chemomechanical caries removal agents: an in vitro study
Source: BMC Oral Health. 2025 Dec 29;26:196. doi: 10.1186/s12903-025-07479-w (PMC12857085; doi:10.1186/s12903-025-07479-w)

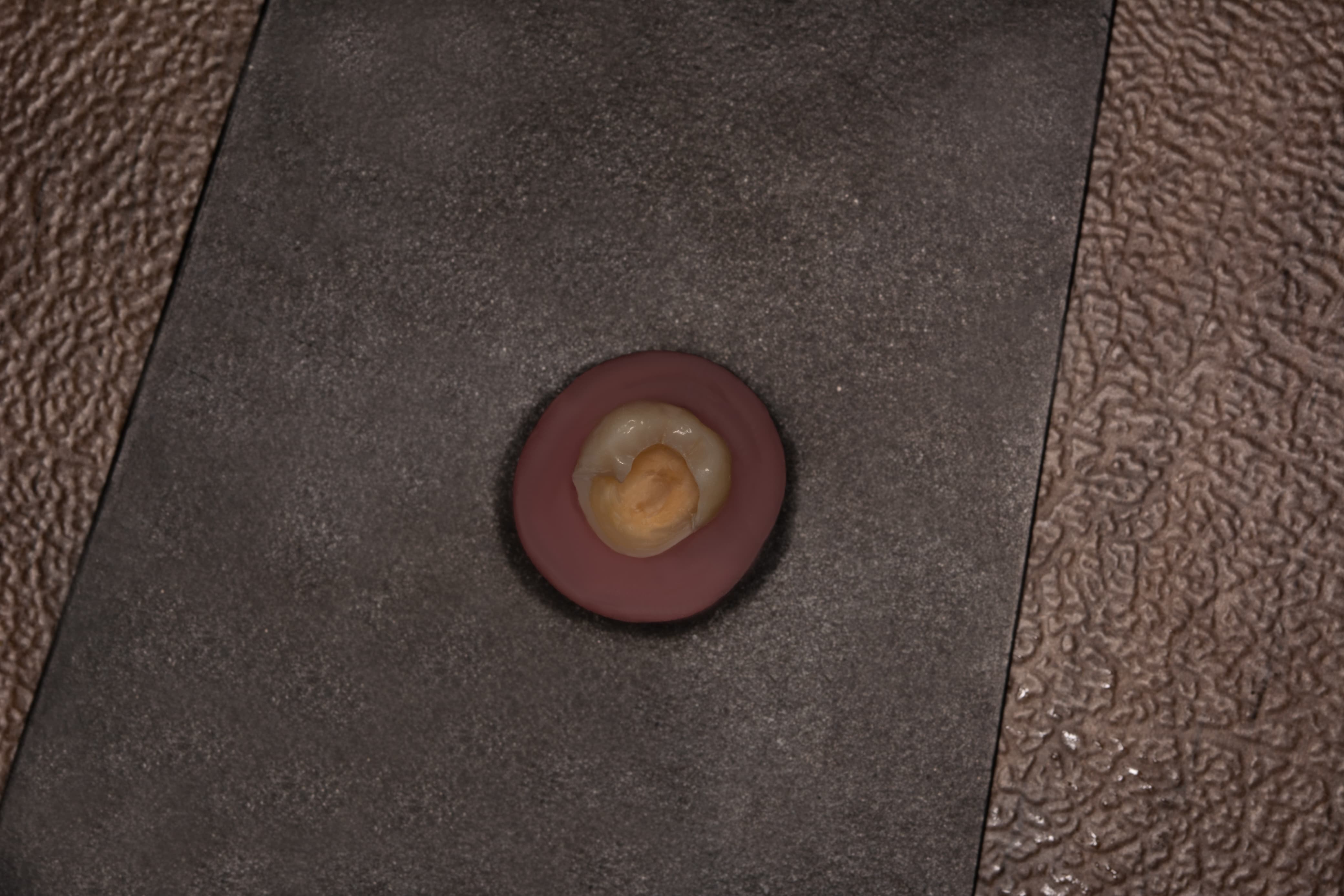

Supplement: Supplementary file 1 — Supplementary Material 1. [file 12903_2025_7479_MOESM1_ESM.zip › Supplementary/Figre 1 d.jpeg]

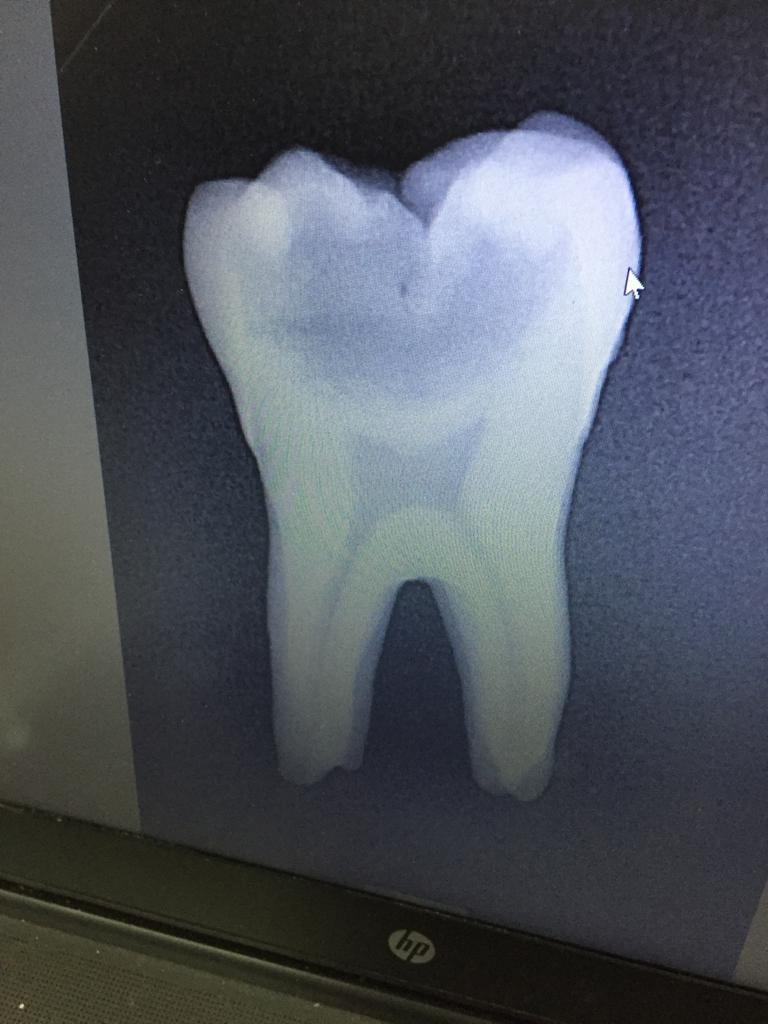

Supplement: Supplementary file 1 — Supplementary Material 1. [file 12903_2025_7479_MOESM1_ESM.zip › Supplementary/Figure 1 a.jpeg]

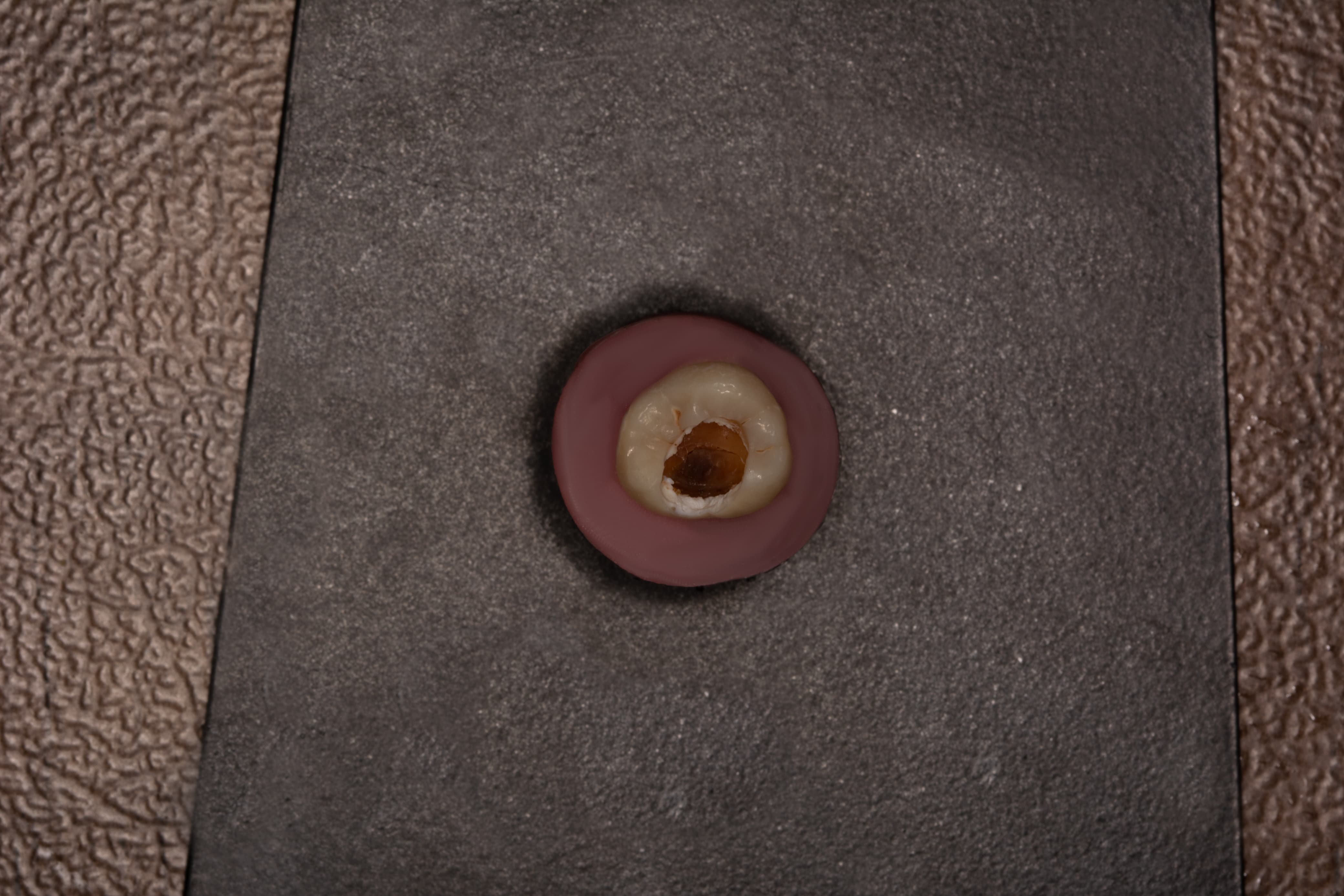

Supplement: Supplementary file 1 — Supplementary Material 1. [file 12903_2025_7479_MOESM1_ESM.zip › Supplementary/Figure 1 b.jpeg]

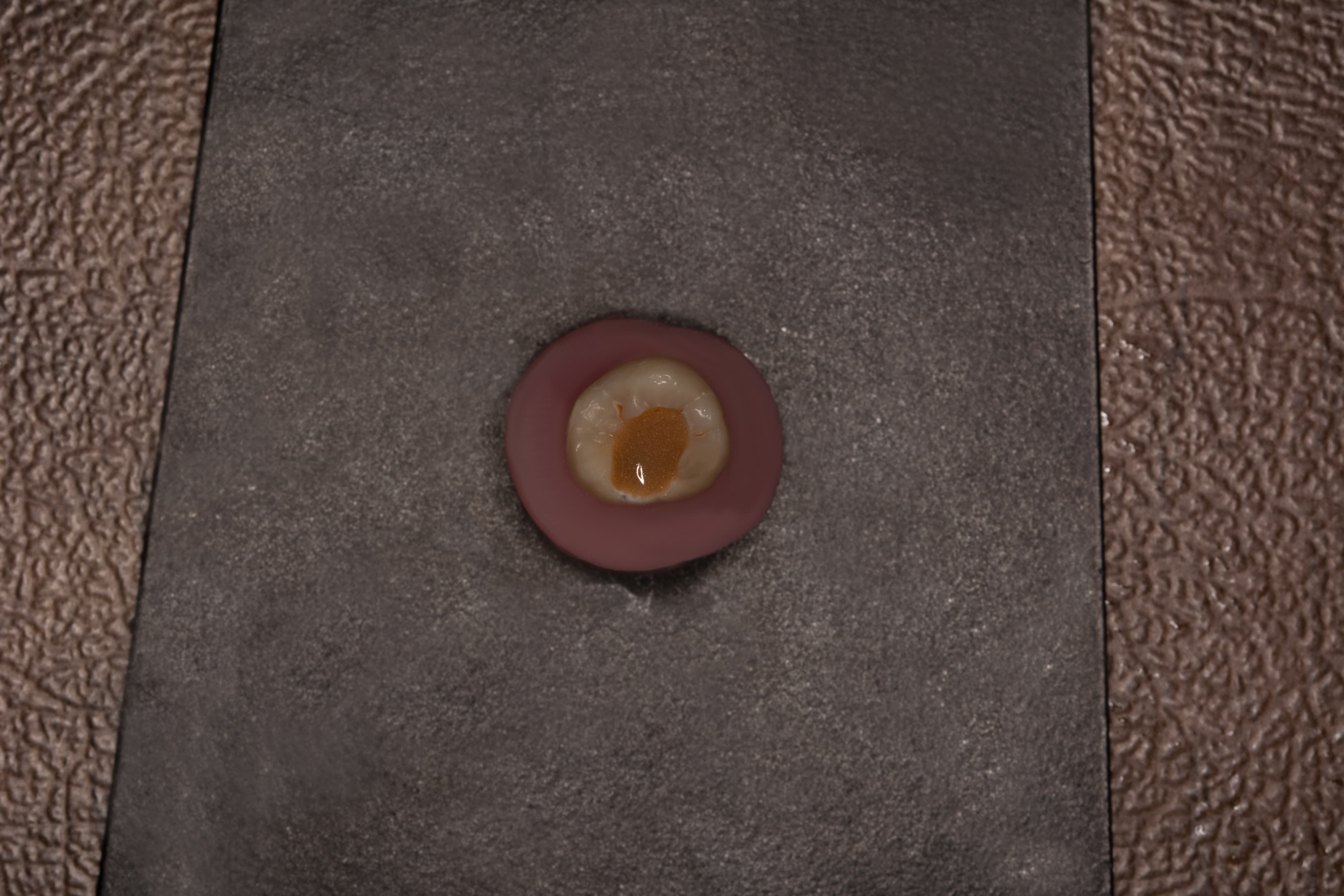

Supplement: Supplementary file 1 — Supplementary Material 1. [file 12903_2025_7479_MOESM1_ESM.zip › Supplementary/Figure 1 c.jpeg]

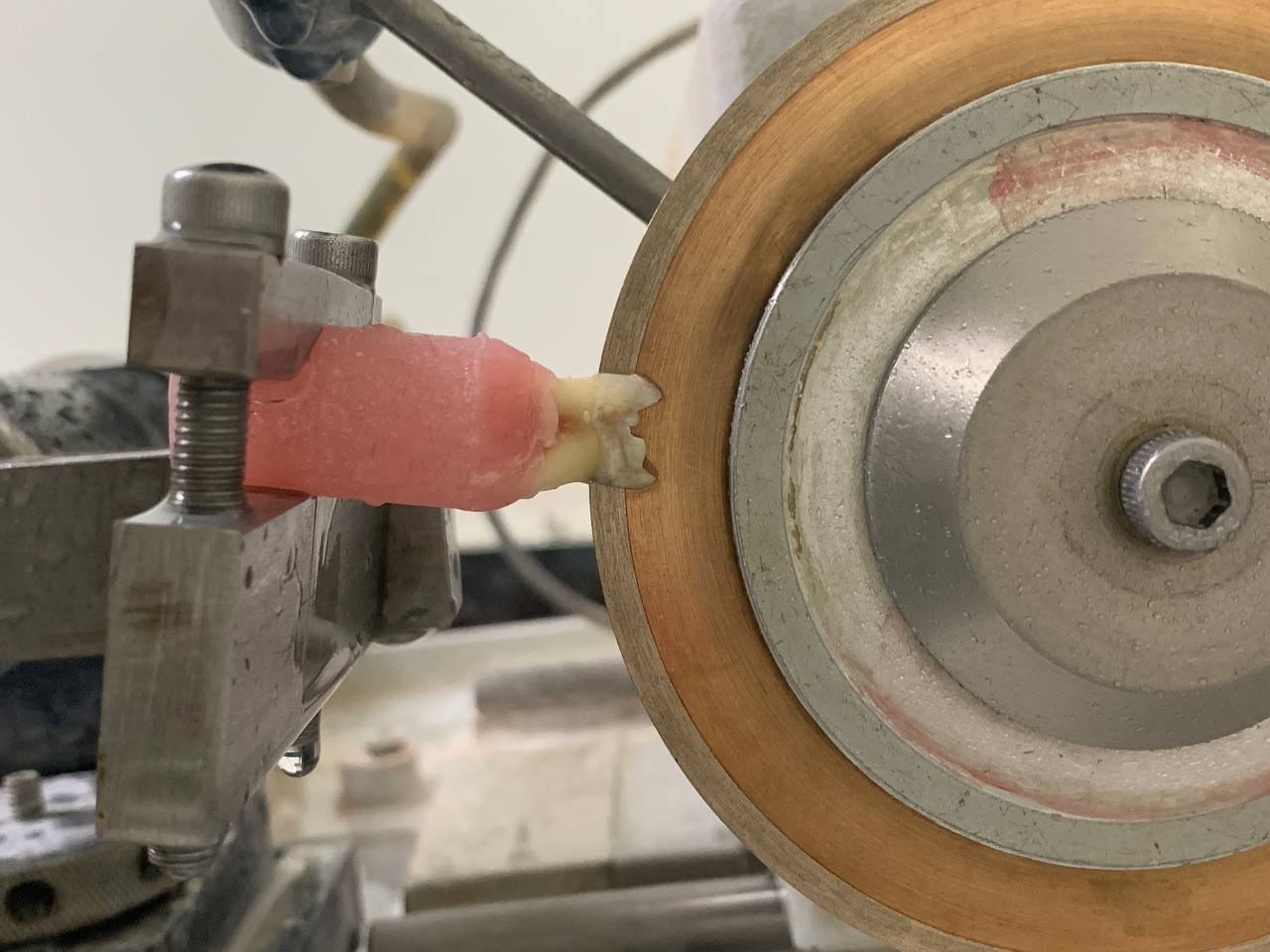

Supplement: Supplementary file 1 — Supplementary Material 1. [file 12903_2025_7479_MOESM1_ESM.zip › Supplementary/Figure 1 e.jpeg]

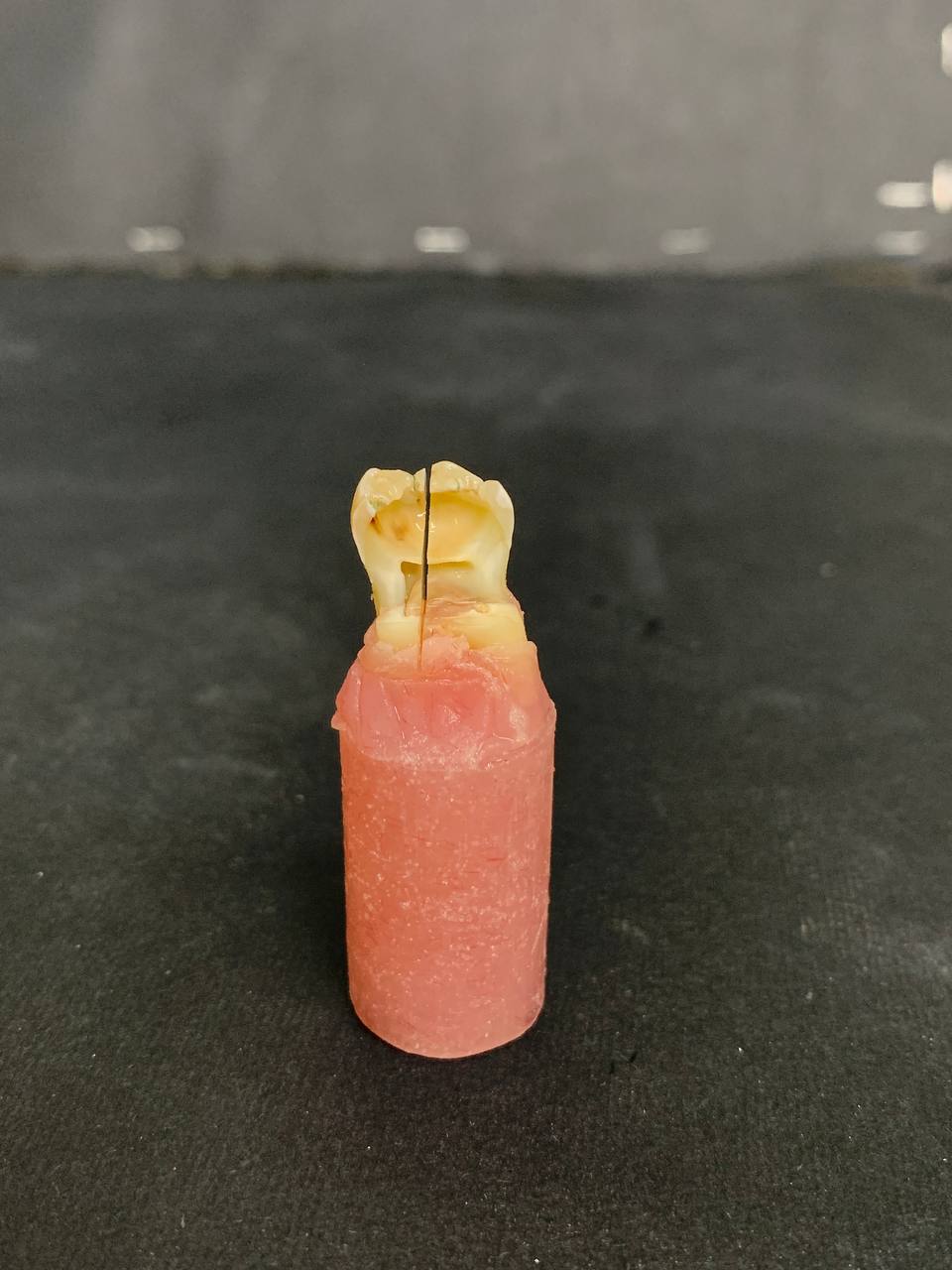

Supplement: Supplementary file 1 — Supplementary Material 1. [file 12903_2025_7479_MOESM1_ESM.zip › Supplementary/Figure 1 f.jpeg]

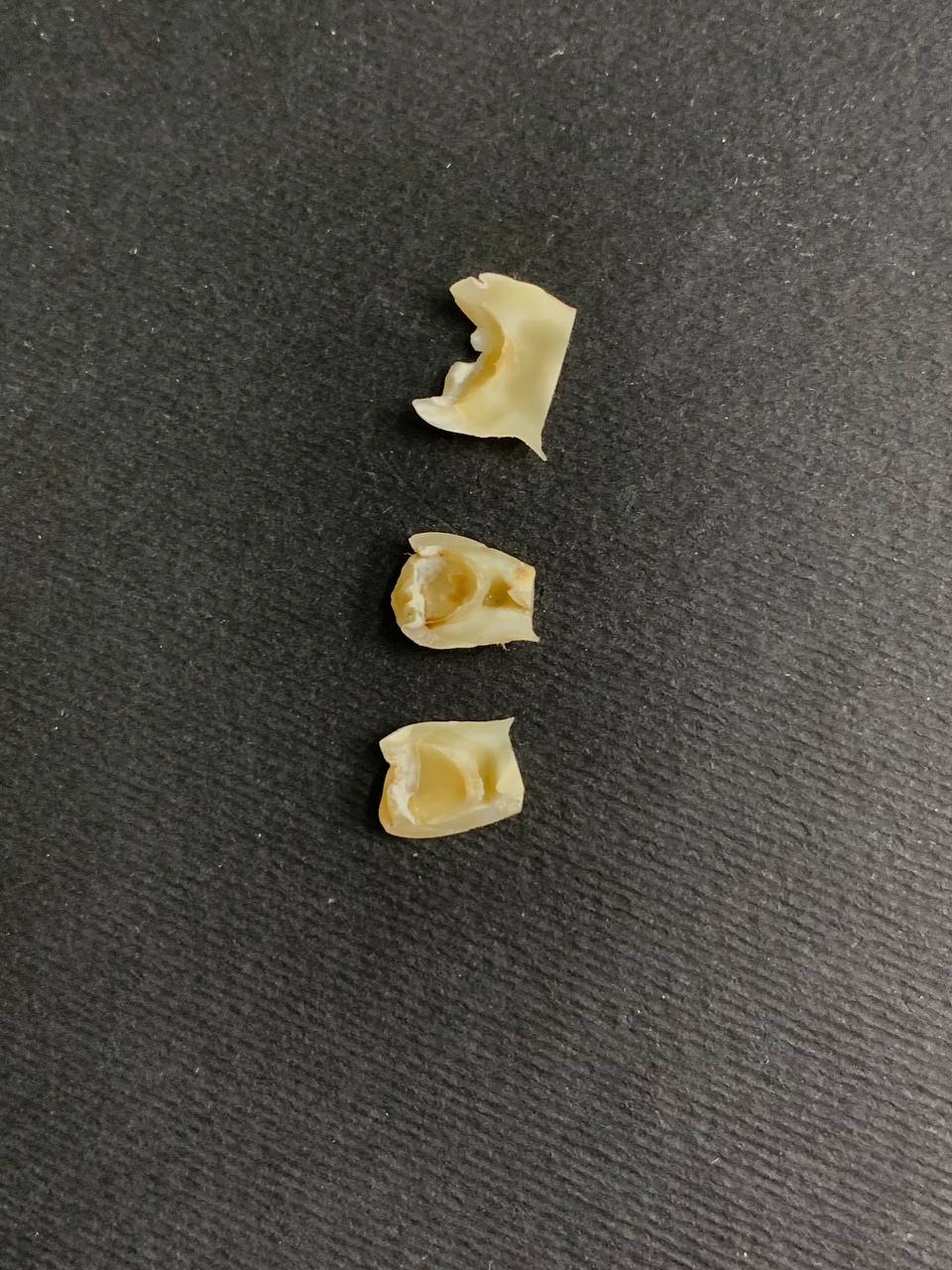

Supplement: Supplementary file 1 — Supplementary Material 1. [file 12903_2025_7479_MOESM1_ESM.zip › Supplementary/Figure 1g.jpeg]

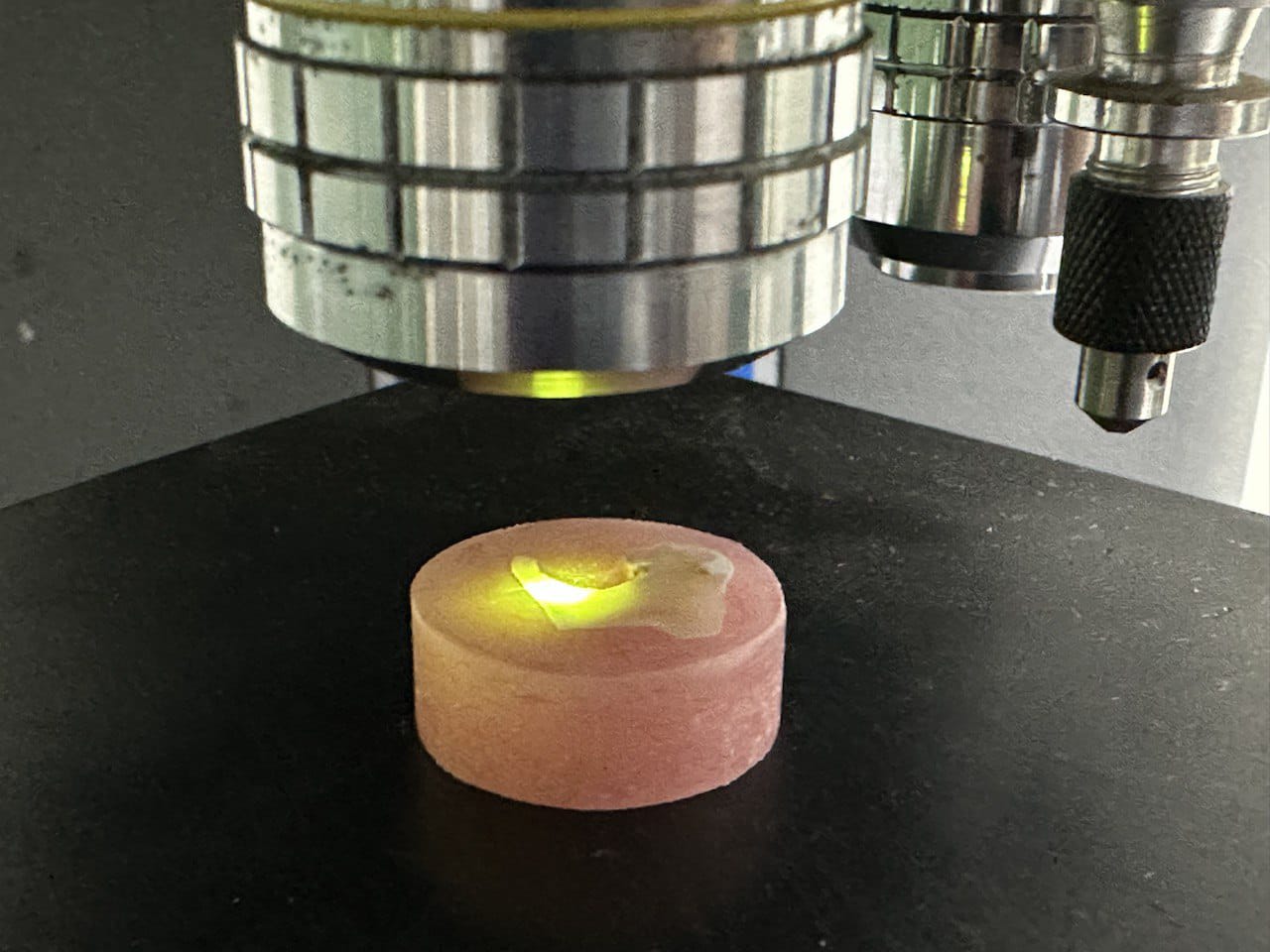

Supplement: Supplementary file 1 — Supplementary Material 1. [file 12903_2025_7479_MOESM1_ESM.zip › Supplementary/figure 2 a.jpeg]

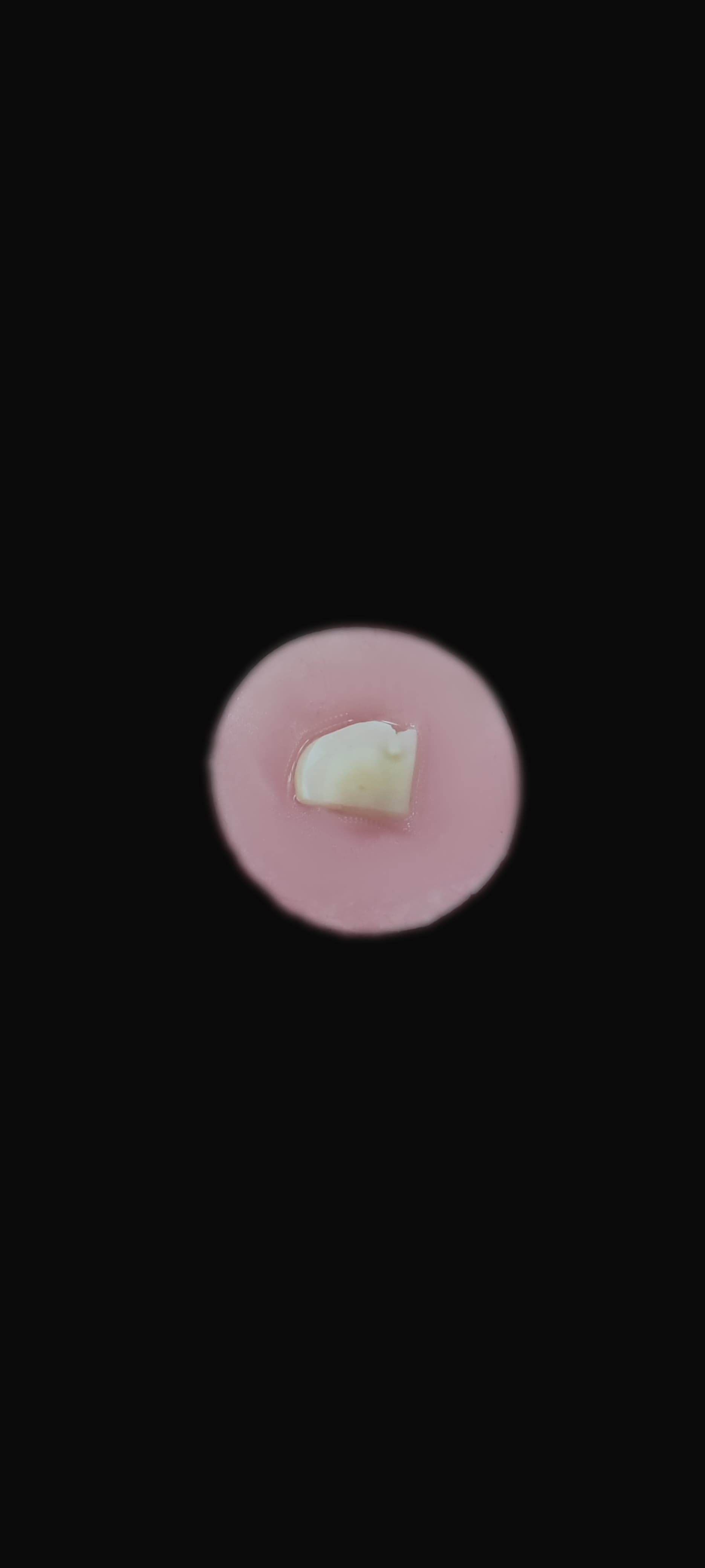

Supplement: Supplementary file 1 — Supplementary Material 1. [file 12903_2025_7479_MOESM1_ESM.zip › Supplementary/figure 2 b.jpeg]

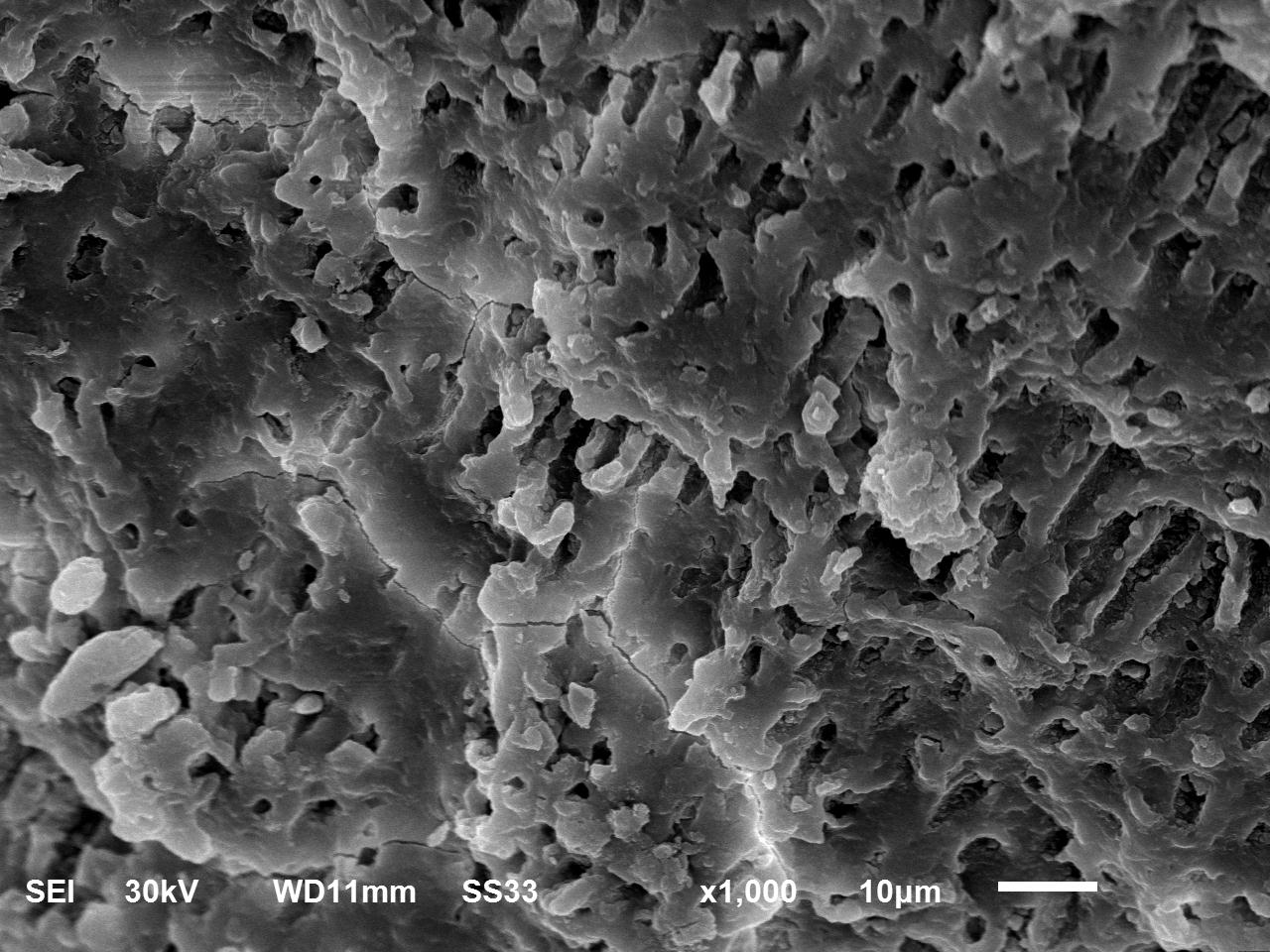

Supplement: Supplementary file 1 — Supplementary Material 1. [file 12903_2025_7479_MOESM1_ESM.zip › Supplementary/figure 3 a.jpeg]

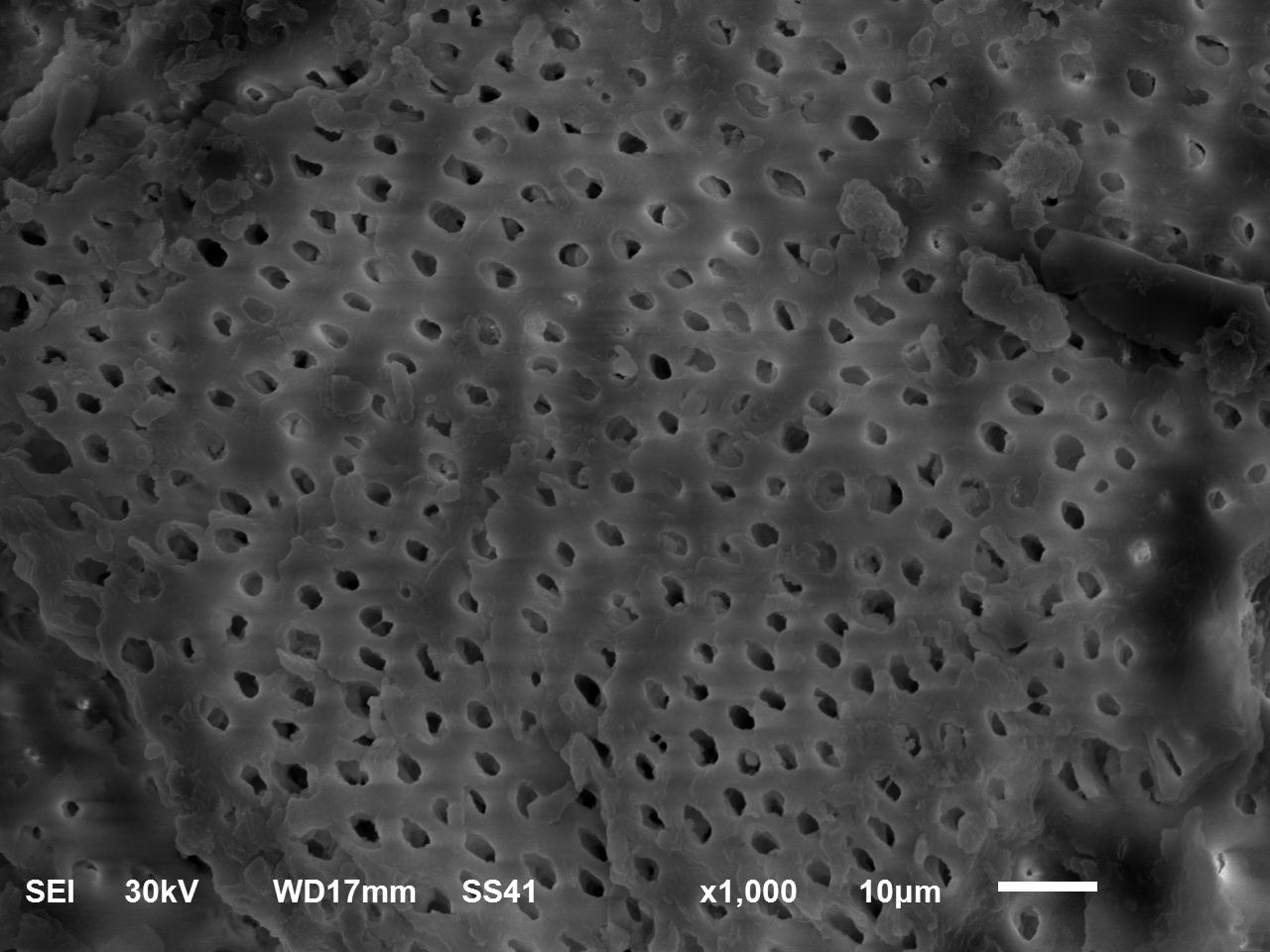

Supplement: Supplementary file 1 — Supplementary Material 1. [file 12903_2025_7479_MOESM1_ESM.zip › Supplementary/figure 3 b.jpeg]

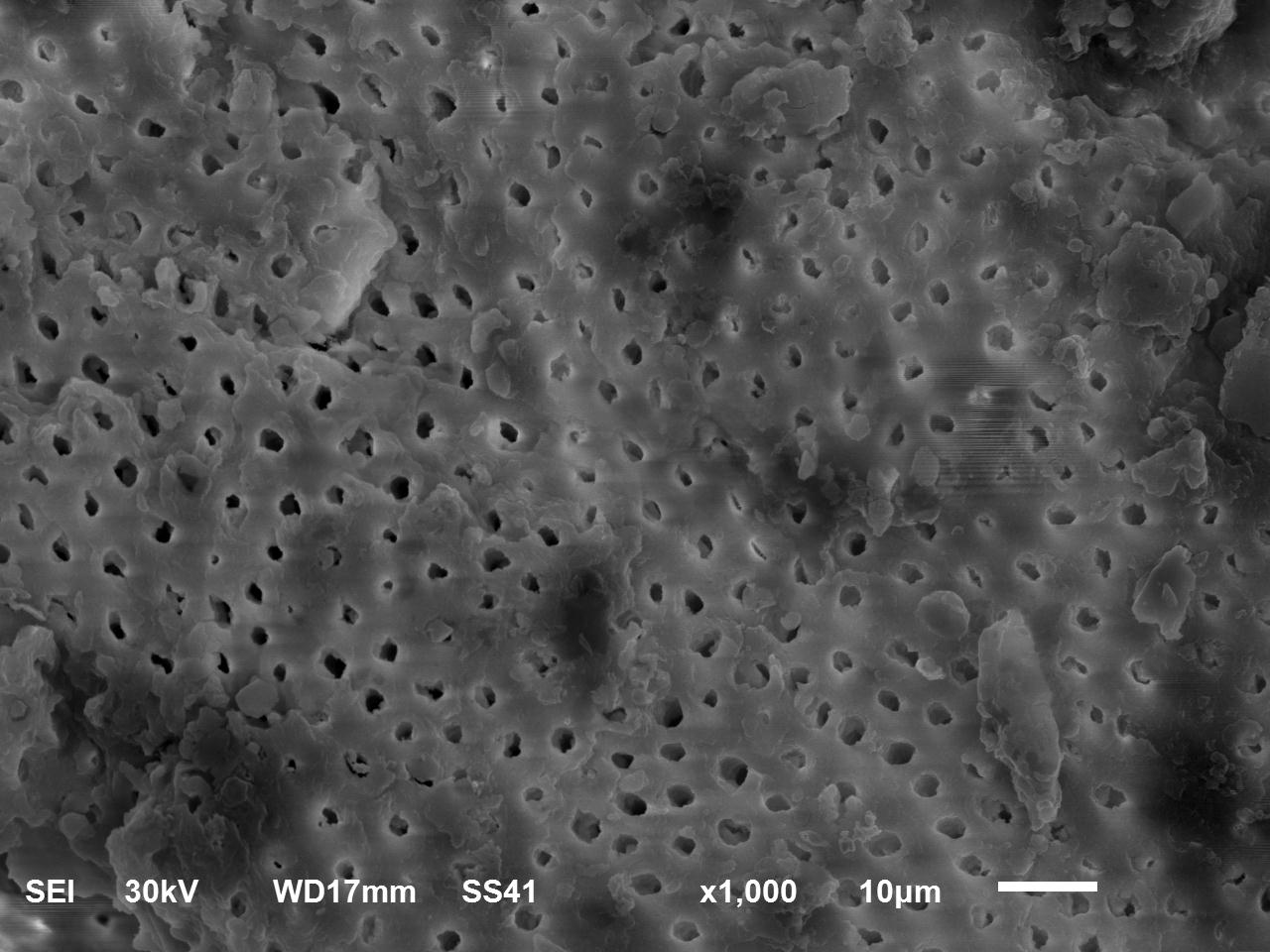

Supplement: Supplementary file 1 — Supplementary Material 1. [file 12903_2025_7479_MOESM1_ESM.zip › Supplementary/figure 3 c.jpeg]

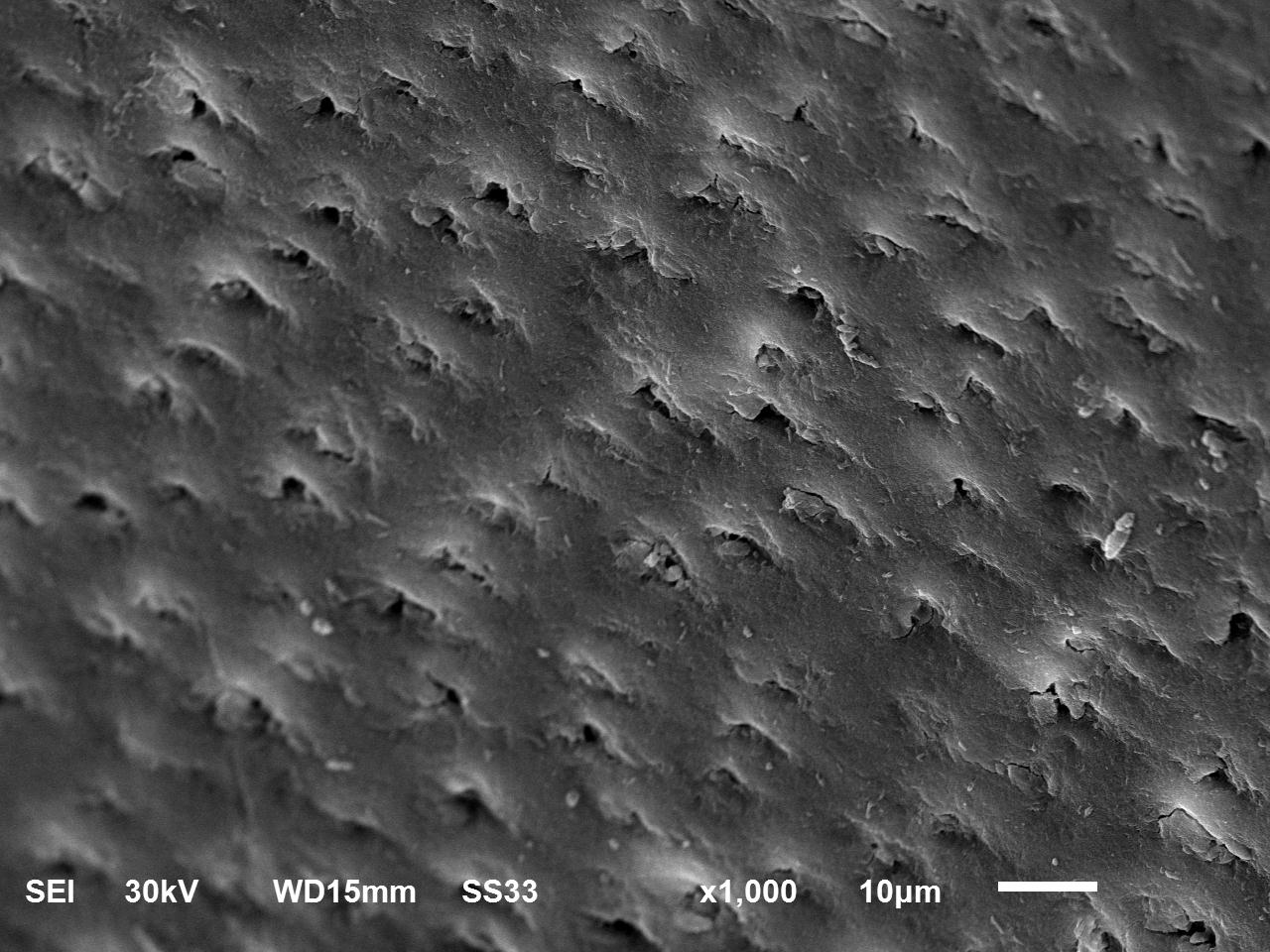

Supplement: Supplementary file 1 — Supplementary Material 1. [file 12903_2025_7479_MOESM1_ESM.zip › Supplementary/Figure 3 d.jpeg]
